# Adenocarcinosarcoma at the esophagogastric junction with long esophageal invasion: a case report

**DOI:** 10.1186/s40792-020-0785-4

**Published:** 2020-01-20

**Authors:** Kenichiro Furukawa, Masahiro Niihara, Takuya Kawata, Shuhei Mayanagi, Yasuhiro Tsubosa

**Affiliations:** 1 Division of Esophageal Surgery, Shizuoka Cancer Center, 1007 Shimonagakubo, Nagaizumi-cho, Sunto-gun, Shizuoka, 411-8777 Japan; 2 Division of Pathology, Shizuoka Cancer Center, 1007 Shimonagakubo, Nagaizumi-cho, Sunto-gun, Shizuoka, 411-8777 Japan

**Keywords:** Esophagus, Esophagogastric junction, Carcinosarcoma, Sarcomatoid carcinoma, Pseudosarcoma, Polypoid sarcoma, Adenocarcinoma

## Abstract

**Background:**

Carcinosarcoma of the esophagus or esophagogastric junction (EGJ) is a rare malignancy with both carcinomatous and sarcomatous components. There is no report of carcinosarcoma arising from the EGJ wherein the carcinomatous element was adenocarcinoma. We describe a patient with carcinosarcoma of the EGJ in which the carcinomatous element was adenocarcinoma.

**Case presentation:**

A 52-year-old man was diagnosed with carcinoma on his EGJ after complaining of appetite loss. All tumor markers (carcinoembryonic antigen, squamous cell carcinoma antigen, alpha-fetoprotein, and carbohydrate antigen 19-9) were within the respective normal ranges. Esophagogastroduodenoscopy showed a 150-mm (100 mm esophageal side and 50 mm gastric side) type 1 tumor on his EGJ. A histopathological examination of a biopsy specimen revealed well-differentiated tubular adenocarcinoma at the gastric side; however, only necrotic tissue was noted on the esophageal side. Contrast-enhanced computed tomography did not reveal any invasion of the adjacent structures; however, it did show five swollen regional lymph nodes. ^18^F-Fluorodeoxyglucose positron emission tomography with computed tomography did not reveal distant metastases. We performed thoracic subtotal esophagectomy, total gastrectomy, and two-field plus left cervical paraesophageal lymphadenectomy. Macroscopically, the lesion consisted of two components: a 7.5-cm type 2 tumor and a 9-cm type 1 tumor at the proximal end of the type 2 tumor. Microscopically, the type 2 tumor showed predominantly solid or cribriform proliferation of tumor cells with clear cytoplasm, which was moderately differentiated adenocarcinoma with enteroblastic-like differentiation. The tumor cells of the adenocarcinoma component had periodic acid-Schiff (PAS)-positive globules and were positive for sal-like protein 4 (SALL 4) and negative for α-fetoprotein (AFP) or human epidermal growth factor receptor type 2 (HER2). The type 1 tumors consisted of the adenocarcinoma-like type 2 tumor and spindle cells (sarcomatous component). Part of the sarcomatous component showed cartilage differentiation. The type 2 and type 1 lesions were continuous lesions. The epicenter of the tumor was located at the EGJ. The adenocarcinoma component was present in 10 of 27 resected lymph nodes. The tumor was diagnosed as carcinosarcoma of the EGJ.

**Conclusions:**

We report a rare patient with carcinosarcoma of the EGJ wherein the carcinomatous element was adenocarcinoma.

## Background

Carcinosarcoma of the esophagus or esophagogastric junction (EGJ) is a rare malignancy with both carcinomatous and sarcomatous components [1–3]. The carcinomatous part is generally squamous cell carcinoma (SCC) [1], and there have been no reports of carcinosarcoma arising from the EGJ wherein the carcinomatous element was adenocarcinoma. We herein report a patient with carcinosarcoma of the EGJ with long esophageal invasion wherein the carcinomatous element was adenocarcinoma.

## Case presentation

A 52-year-old man was diagnosed with carcinoma on his EGJ following complaints of appetite loss. A histopathological examination of a biopsy specimen revealed adenosquamous carcinoma at a previous hospital. He was then referred to our hospital.

He had undergone appendectomy due to appendicitis at 13 years of age. He was an ex-smoker and drank 360 ml of distilled spirits every day. His height and weight were 178.1 cm and 51.9 kg, respectively. His body temperature was elevated to 38.5 °C. A hematologic examination showed inflammatory reaction (white blood cell 12430/μL, neutrophil cell 86%, and C-reactive protein 4.80 mg/dL), mild renal disorder (blood urea nitrogen 26.8 mg/dL and creatinine 1.07 mg/dL), and alkaline phosphatase elevation (361 U/L). All tumor markers (carcinoembryonic antigen, squamous cell carcinoma antigen, alpha-fetoprotein, and carbohydrate antigen 19-9) were within the respective normal ranges. Bacterial cultivation of the blood, sputum, and urine showed no obvious infectious findings.

**Fig. 1:**
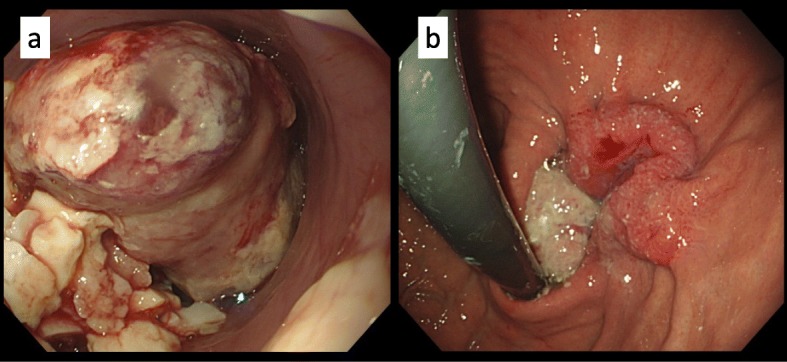
Findings of esophagogastroduodenoscopy. **a** Esophageal side, **b** gastric side. Esophagogastroduodenoscopy showed a 150-mm (100 mm esophageal side and 50 mm gastric side) type 1 tumor at the esophagogastric junction obstructing the passage of food. The epicenter of the tumor appeared to be on the esophagus

Esophagogastroduodenoscopy showed a 150-mm (100 mm esophageal side and 50 mm gastric side) type 1 tumor on his EGJ that was obstructing the passage of food (Fig. 1). The epicenter of the tumor appeared to be on the esophagus. A histopathological examination of a biopsy specimen revealed well-differentiated tubular adenocarcinoma at the gastric side; however, only necrotic tissue was observed at the esophageal side. Endoscopically, there was no evidence of Barrett’s esophagus.

Contrast-enhanced computed tomography did not reveal any invasion of the adjacent structures; however, five swollen regional lymph nodes were detected. ^18^F-Fluorodeoxyglucose positron emission tomography with computed tomography did not reveal distant metastases. We clinically diagnosed his tumor as advanced esophageal cancer, Lt Ae G, type 1, 150 mm, well-differentiated tubular adenocarcinoma, cT3 N2 M0, cStage IIIB according to the Union for International Cancer Control TNM classification of malignant tumors, seventh edition (UICC-TNM 7th).

We performed video-assisted thoracic subtotal esophagectomy, open total gastrectomy, two-field plus left cervical paraesophageal lymphadenectomy, subcutaneous reconstruction using a free jejunal graft, and jejunostomy. The operation time was 8 h and 35 min, and the estimated blood loss was 64 mL. He experienced postoperative complications of grade IIIb anastomotic leakage, grade II pneumonitis, and grade I left recurrent laryngeal nerve paralysis according to the Clavien–Dindo classification [4], all of which improved with treatment, and he was discharged on post-operative day 24.

**Fig. 2:**
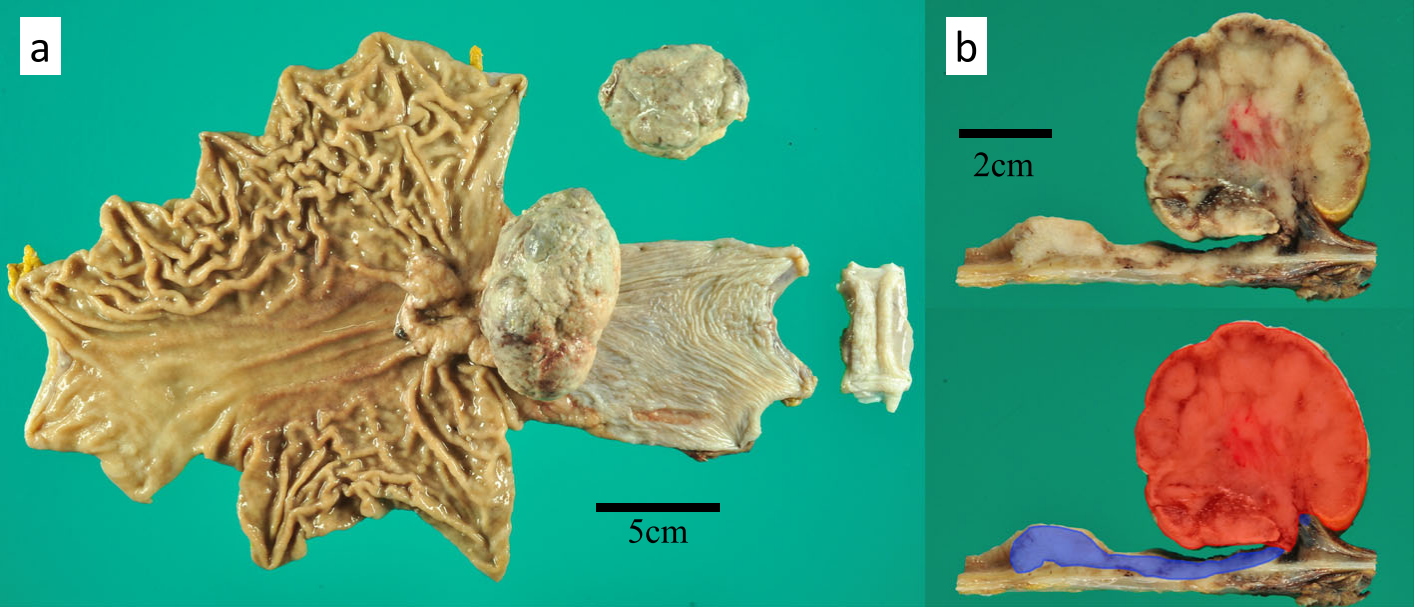
Macroscopic findings of the resected specimen and distribution of the carcinomatous and sarcomatous components. **a** The lesion consisted of two components: a 7.5-cm type 2 tumor and a 9-cm type 1 tumor at the proximal end of the type 2 tumor. We also observed a 6-cm mass of tumor tissue that appeared to be separate from the type 1 tumor. **b** The type 2 tumor showed a white-gray cut surface, while the type 1 tumor showed the same white-gray cut surface with necrosis and hemorrhaging. The blue areas indicate the carcinomatous component, and the red area shows the carcinomatous with sarcomatous components

**Fig. 3:**
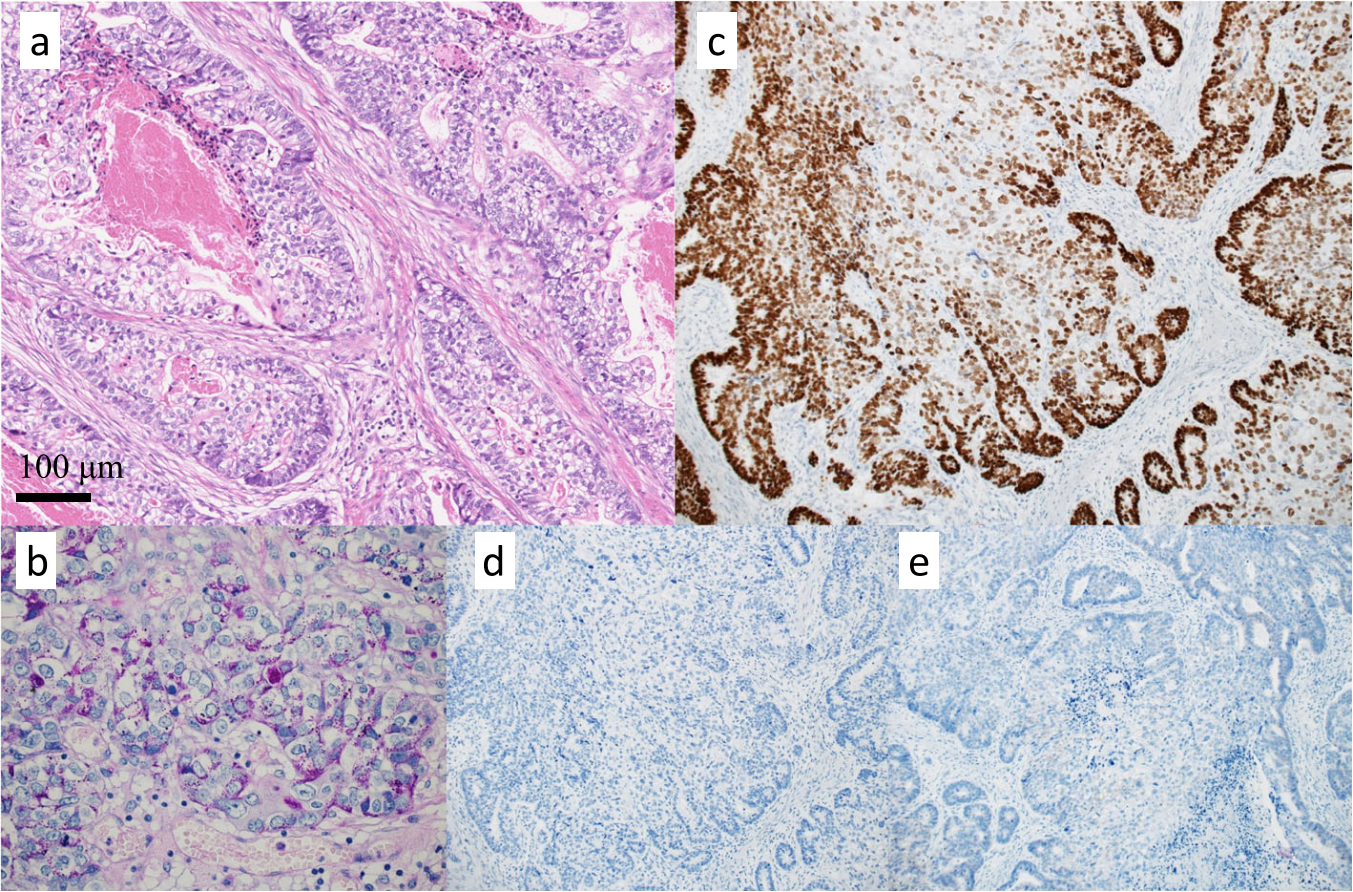
Microscopic findings of the type 2 tumor. **a** The type 2 tumor predominantly showed solid or cribriform proliferation of tumor cells with clear cytoplasm, which was moderately differentiated adenocarcinoma with enteroblastic-like differentiation (hematoxylin-eosin staining). The tumor cells of the adenocarcinoma component had periodic acid-Schiff (PAS)-positive globules (**b**) and were positive for sal-like protein 4 (SALL 4) (**c**) and negative for α-fetoprotein (AFP) (**d**) and human epidermal growth factor receptor type 2 (HER2) (**e**)

**Fig. 4:**
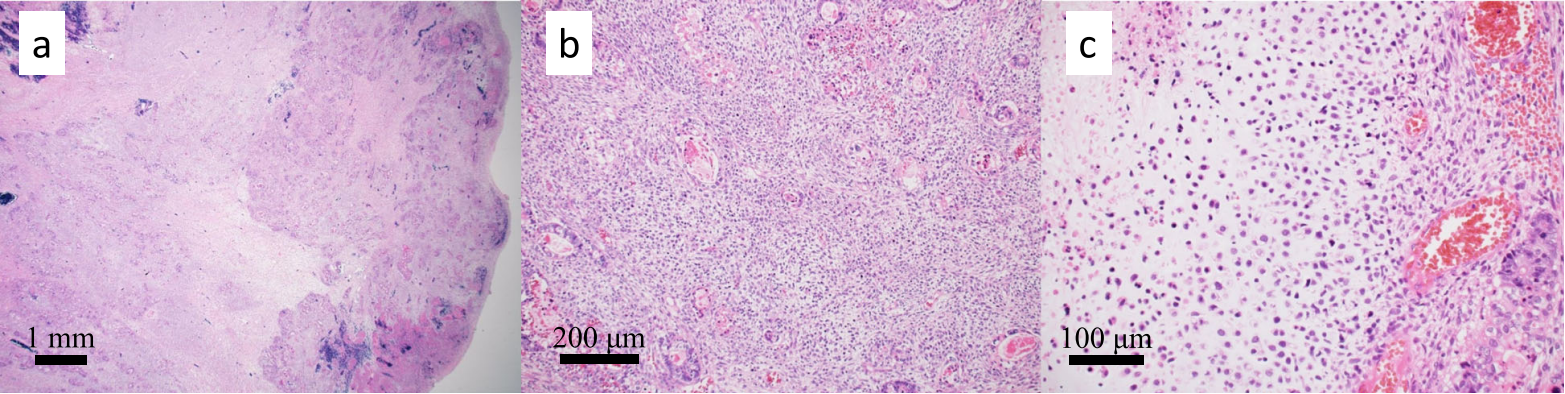
Microscopic findings of the type 1 tumors. Hematoxylin-eosin staining. **a**, **b** The type 1 tumors consisted of adenocarcinoma, like the type 2 tumor, and spindle cells. **c** Part of the sarcomatous component showed cartilage differentiation

Macroscopically, the lesion consisted of two components: a 7.5-cm type 2 tumor and a 9-cm type 1 tumor at the proximal end of the type 2 tumor (Fig. 2a). The type 2 tumor showed a white-gray cut surface, while the type 1 tumor showed the same white-gray cut surface with necrosis and hemorrhaging (Fig. 2b). Whether the epicenter of the lesion was at the gastric side or esophageal side was unclear, as most of the EGJ had been replaced by the tumor. Microscopically, the type 2 tumor was predominantly solid or cribriform proliferation of tumor cells with clear cytoplasm, which was moderately differentiated adenocarcinoma with enteroblastic-like differentiation (Fig. 2b, Fig. 3a). The tumor cells of the adenocarcinoma component had periodic acid-Schiff (PAS)-positive globules and were positive for sal-like protein 4 (SALL 4) and negative for α-fetoprotein (AFP) or human epidermal growth factor receptor type 2 (HER2) (Fig. 3b–e). The reasons why enteroblastic-like differentiation was considered to have occurred were as follows: the tumor cells had clear cytoplasm, while they were also PAS-positive and SALL 4-positive. The type 1 tumors consisted of adenocarcinoma, like the type 2 tumor, which was positive for SALL 4, and spindle cells (sarcomatous component) growing mainly in the submucosal layer (Fig. 2b, Fig. 4a, b). There was no obvious SCC component in the type 1 tumor. Part of the sarcomatous component showed cartilage differentiation (Fig. 4c). The type 2 and type 1 lesions were continuous lesions, and there were smooth transitional features between the two lesions. The epicenter of the tumor was at the EGJ. The deepest invasive site was associated with the type 2 tumor, which showed invasion to the subserosa. The horizontal and vertical margins were negative for tumor cells. Histologically, there was no evidence of Barrett’s esophagus in the resected specimen. We observed all resected esophagus specimens with a cutting width of approximately 10 mm. The adenocarcinoma component was present in 10 of 27 resected lymph nodes. The tumor was finally diagnosed as carcinosarcoma of the EGJ with a carcinomatous component of adenocarcinoma, Siewert type II, type 1 + 2, 110 mm, pT3 N3 M0, pStage IIIC according to UICC-TNM 7th.

The patient underwent adjuvant systemic chemotherapy with S-1 for gastric adenocarcinoma but developed para-aortic lymph node metastases 5 months after surgery.

## Discussion

Carcinosarcoma of the esophagus is rare and is also referred to as spindle cell carcinoma, sarcomatoid carcinoma, pseudosarcomatous SCC, polypoid carcinoma, metaplastic carcinoma, SCC with spindle-cell component, and carcinoma with mesenchymal stroma [5, 6]. The incidence of such tumors has been reported to be 0.4 to 2.4% among all esophageal neoplasms [5, 7–10].

The carcinomatous part of esophageal carcinosarcoma generally consists of an SCC [1]. We found only two patients reported as having carcinosarcoma of esophagus wherein the carcinomatous component was adenocarcinoma [1, 8]. Dworak reported a patient with carcinosarcoma in Barrett’s esophagus [1], and Zhang reported 71 patients with esophageal carcinosarcoma in which one patient had a carcinomatous component of adenocarcinoma [8]. In our patient, there was no obvious SCC component.

Carcinosarcoma of the EGJ is extremely rare. We found only one case report of carcinosarcoma arising from the EGJ [3]. The patient was diagnosed with carcinosarcoma because the tumor cells were positive for both cytokeratin and vimentin. The type of carcinoma was unknown because the tumor cells were a single and uniform population of spindle-shaped cells, and immunohistochemical staining was not performed to determine the carcinoma type. Therefore, this is the first report describing carcinosarcoma of the EGJ whose carcinomatous component was adenocarcinoma. Furthermore, although most cases of adenocarcinoma of the EGJ arise from Barrett’s esophagus [11, 12], there was no evidence of Barrett’s esophagus endoscopically or histologically in our patient.

In general, esophageal carcinosarcoma presents as an intraluminal polypoid mass located in the middle and lower thoracic esophagus. Therefore, if a polypoid tumor is observed on EGJ and the result of a biopsy is not typical adenocarcinoma or SCC, we should consider the possibility of carcinosarcoma.

The polypoid appearance results in an early onset of symptoms and thus an earlier diagnosis and treatment. Although esophageal carcinosarcoma is more likely to be diagnosed at an early T stage, it carries a highly aggressive potential for lymph node metastasis [7, 8, 13]. The carcinomatous component metastasizes to the lymph node more often than the sarcomatous component [7]. In our patient, unfortunately, the carcinosarcoma invaded toward the adventitia, and only the carcinomatous component was found to have metastasized to resected lymph nodes, as in previous report.

Immunohistochemical staining can help clarify how carcinosarcoma develops. Specifically, it appears that adenocarcinoma with enteroblastic-like differentiation differentiated to mesenchymal cells, including cartilage cells, the present case.

The tumor in our patient may have been gastric cancer with esophageal invasion, as most of the EGJ had been replaced by the tumor, and the epicenter of the tumor was unclear. Regarding gastric carcinosarcoma, there have been 38 patients reported in the English literature [14–46]. Among them, there was only one patient whose tumor had invaded the esophagus and no patients whose tumor has enteroblastic-like differentiation [39]. However, some reports did not mention the extent of esophageal invasion or details concerning the carcinoma component.

## Conclusion

We herein report a rare case of carcinosarcoma of the EGJ with long esophageal invasion wherein the carcinomatous element was adenocarcinoma.

## Data Availability

Not applicable

## Abbreviations

EGJ: Esophagogastric junction
SCC: Squamous cell carcinoma
UICC-TNM 7th: Union for international cancer control TNM classification of malignant tumors seventh edition
